# Predictive modeling for cervical cancer: existing AI approaches and the emerging role of vaginal microbiome

**DOI:** 10.3389/fnetp.2026.1799486

**Published:** 2026-06-05

**Authors:** Michelle Gomes, Zonglun Li, Adeola Olaitan, Aleksandra Gentry-Maharaj

**Affiliations:** 1 Department of Global Health and Development, The London School of Hygiene and Tropical Medicine (LSHTM), London, United Kingdom; 2 Anne’s Day Ltd. (Daye), London, United Kingdom; 3 Department of Women’s Cancer, EGA Institute for Women’s Health, University College London, London, United Kingdom; 4 Wolfson Institute of Population Health, Queen Mary University of London, London, United Kingdom

**Keywords:** biomarkers, cervical cancer, clinical validation, equity, multi-omics, network physiology, predictive models, risk stratification

## Abstract

Cervical cancer remains a major global health burden, yet current screening tools lack precision in identifying which women with high-risk human papillomavirus (HPV) infection will progress to high-grade lesions or cancer. Within a network-physiology framework, cervical carcinogenesis is viewed as emerging from dynamic interactions between viral dynamics, host immunity, vaginal ecology, vaccination status and behaviour rather than from isolated risk factors. This perspective review examines artificial intelligence (AI) approaches for cervical cancer prediction and evaluates the emerging role of the vaginal microbiome as a complementary biomarker within these interconnected physiological networks. The review synthesises evidence linking non-Lactobacillus-dominated or *Lactobacillus* iners-rich vaginal communities with increased HPV persistence and cervical intraepithelial neoplasia, contrasted with protective *Lactobacillus* crispatus-dominant communities, and outlines how these ecological signatures could be combined with HPV genotype and clinical factors in multi-modal models. A structured narrative synthesis of published AI tools demonstrates that current prognostic, diagnostic and screening algorithms rely mainly on demographic, clinical or imaging variables, with no validated models yet integrating vaginal microbiome profiles into cervical cancer risk calculators. The manuscript proposes a technical framework for microbiome-enabled modelling, covering feature engineering from community state types, algorithm selection, handling of high-dimensional omics data, and staged validation in NHS-relevant populations. Finally, it outlines a translational pathway for embedding microbiome-informed risk models into cervical screening using self-collected tampon sampling, AI-driven triage and digital decision support, and identifies key unmet needs, including longitudinal multi-omic cohorts, international consortia and robust bias auditing.

## 1 Introduction

Cervical cancer remains a major health concern worldwide, with persistent infection by high-risk human papillomavirus (HPV) types as the cause, yet predicting which infections will progress to high-grade lesions or invasive cancer remains challenging (Hakim et al., 2025; Hall et al., 2025; Gopalkrishnan and Karim, 2025). Understanding which women are at real risk is essential for effective prevention, and emerging evidence indicates that this risk is best understood through patterns in multi-omic networks rather than isolated biomarkers (Jafari et al., 2024; Gisca et al., 2025; Hu and Ma, 2018). Integrating genomics, transcriptomics, proteomics, methylation data, and microbiome profiles can uncover network markers that more accurately predict progression than single factors, supporting a shift toward multi-modal data integration in clinical research and care (Gisca et al., 2025; Martínez-Rodríguez et al., 2021; Multi-omics approaches in cancer research, 2021). Within this framework, HPV infection, host immune responses, and vaginal microbiome composition interact in a broader physiological network, where disease mechanisms and progression emerge from these interactions rather than from additive individual risks (Schellekens et al., 2025; Papamentzelopoulou et al., 2025; Alizhan et al., 2025). Meta-analyses and multi-omics studies consistently link non-*Lactobacillus*-dominated or *Lactobacillus iners*–rich communities to higher risks of HPV persistence and cervical disease, while *Lactobacillus crispatus*–dominant microbiomes appear protective, likely through effects on local immunity, tissue integrity, and viral clearance (Carter et al., 2023; Wang et al., 2019; Brusselaers et al., 2019). A key challenge is to identify and validate biomarkers that capture patterns across these genomic, proteomic, and microbiome layers and to translate such network markers into clinically usable tools (Naikoo et al., 2025). Although advances in artificial intelligence (AI) have transformed cancer risk prediction in other domains, most published algorithms for cervical cancer still rely on clinical features and HPV genotype alone, with limited incorporation of multi-omic or microbiome data. This perspective review therefore synthesises existing AI approaches and outlines the conceptual and technical requirements for developing multi-modal predictive models that explicitly integrate vaginal microbiome information into cervical cancer screening, diagnosis, and prognosis (Gisca et al., 2025; Abrar et al., 2025).

AI models for cervical cancer prediction can be grouped into three broad use cases: screening and triage, diagnostic classification, and prognosis or survival prediction (Abrar et al., 2025; Zhang et al., 2025; Dellino et al., 2024). In the current literature, most AI models for cervical cancer operate on either tabular clinical data or imaging data, but they do not map neatly onto real-world categories of “screening” versus “diagnosis”, which in practice are longitudinal and tightly coupled (The Role of Artificial Intelligence; Ali et al., 2025; Ramírez et al., 2025). Tabular models most often use logistic regression, random forests, support vector machines or gradient boosting to classify risk or predict outcomes from demographic, clinical and HPV variables, whereas imaging-based models largely rely on convolutional neural networks and related deep learning architectures to distinguish normal, low-grade and high-grade lesions on Pap smears, colposcopy or cervicography (Al Mudawi and Alazeb, 2022; Sun et al., 2022). Across these studies there is substantial overlap in intended use, with some CNN-based systems applied in settings closer to screening or triage, and very few tools explicitly designed for longitudinal, programme-level screening as implemented in the NHS. Prognostic and survival models have mostly used Cox proportional hazards models, Weibull models, or tree based survival methods trained on demographics, tumour stage, histology and treatment variables to predict time to recurrence or death. Each class of method offers different trade-offs: some classical statistical models provide clear effect estimates and are well suited to modest sized, tabular datasets, whereas deep neural networks excel on high dimensional imaging data but may require larger samples and more careful regularisation and explainability strategies (Pourakbar et al., 2024; colleagues, 2024; Mitra et al., 2015).

This article is a structured narrative review and perspective rather than a full systematic review. We identified relevant primary studies and reviews on AI-based cervical cancer prediction, HPV self-sampling, and vaginal microbiome HPV interactions through targeted searches in PubMed and Google Scholar, using combinations of terms such as ‘cervical cancer’, ‘prediction model’, ‘artificial intelligence’, ‘machine learning’, ‘vaginal microbiome’, ‘community state type’, and ‘self-sampling’, with emphasis on publications from 2015 onwards and recent landmark papers up to January 2026. Reference lists of key systematic reviews and narrative overviews were screened to identify additional studies. Because our aim was to synthesise methodological trends and outline a translational framework, we did not perform a formal systematic review with PRISMA-based study selection or meta-analysis.

## 2 Epidemiology and natural history of HPV

Worldwide, cervical cancer affects about 660,000 women annually and leads to over 350,000 deaths, ranking as the fourth most common cancer in women. In the United Kingdom there are approximately 3,200 new cases and 850 deaths each year, with the highest incidence in women aged 30–34 (World Health Organization, 2024). Persistent infection with high-risk HPV genotypes 16 and 18 accounts for roughly 70% of invasive cancers, with other types such as HPV 31, 33, 45, 52, and 58 increasingly recognised in precancerous and malignant lesions (Luo et al., 2023; National Cancer Institute, 2022). HPV infection typically involves initial acquisition and acute replication, and most infections clear within one to 2 years through effective host immune responses; however, persistence of the same high-risk HPV type for at least 12 months sharply increases the risk of high-grade cervical intraepithelial neoplasia (CIN2/3) and invasive cancer (IARC Working Group, 2007; Bruni, 2025). Persistence is driven by a combination of host and environmental factors, including immunosuppression, smoking, and hormonal contraceptive use, yet outcomes vary among women with similar exposures, indicating that persistent HPV infection alone does not fully explain progression (Hewavisenti et al., 2023). Current data point toward genetic susceptibility and the composition of the cervicovaginal microbiome as important influences on HPV persistence and progression, and persistent infection and its sequelae are shaped by host, viral, and microenvironmental factors, including the vaginal microbiome (Alizhan et al., 2025; Bautista et al., 2025; Lebeau et al., 2022).

The burden of cervical cancer remains disproportionately concentrated in low- and middle-income countries (LMICs), where over 80%–90% of cases and deaths occur and screening coverage is often low despite WHO elimination targets (World Health Organization, 2024). In these settings, scalable risk-stratification tools and HPV self-sampling strategies have the potential to improve uptake and cost-effectiveness, particularly when implemented as part of integrated ‘screen-and-treat’ programmes rather than resource-intensive multi-visit pathways.

HPV vaccination has begun to reshape the risk landscape, with substantial reductions in high-grade cervical lesions observed in vaccinated cohorts, but its impact is heterogeneous across age groups, geographies, and screening histories (Harper et al., 2025). In the United Kingdom, school-based vaccination programmes now cover multiple birth cohorts, creating a growing proportion of women who enter cervical screening with partial or complete protection against vaccine-covered HPV genotypes, while older women, late adopters, migrants and those from underserved communities remain disproportionately unvaccinated (Beer et al., 2014; GOV, 2025). HPV vaccination status therefore represents a critical modifier of underlying risk and of the pre-test probability of high-risk HPV and CIN, and should be explicitly incorporated into future multi-modal prediction models alongside HPV genotype, cytology, microbiome composition and behavioural factors (Galani et al., 2025).

In England, the NHS Cervical Screening Programme invites women and people with a cervix aged 25–64 for primary HPV screening at intervals of three to 5 years, with around 4.6–5.2 million invitations issued and approximately 3.2–3.4 million tests completed each year (England, 2025). Most attendees test HPV negative and are returned to routine recall, whereas those who are HPV positive undergo reflex cytology and, if abnormalities or persistent HPV are detected, are referred for colposcopy to detect and treat CIN2/3 and early cancer (England, 2025; Public Health England, 2024). Although primary HPV screening has improved sensitivity and reduced cancer incidence compared with cytology-based programmes, a substantial proportion of screened individuals will have transient HPV infection but never develop CIN2+, leading to large numbers of follow-up tests and colposcopies for a relatively small number of high-grade lesions (Public Health England, 2024). At present, clinical practice lacks reliable tools beyond HPV genotype and cytology to distinguish, among HPV-positive women, those at highest risk of progression to CIN2/3 and cancer from those likely to clear infection, underscoring the need for integrated risk assessment approaches that can incorporate additional biological layers such as the vaginal microbiome (Kamzayeva et al., 2025; Hakim et al., 2025). Within this context, vaginal microbiome profiling has the potential to refine risk prediction and triage by identifying dysbiotic signatures associated with persistent high-risk HPV and CIN2/3, thereby reducing unnecessary surveillance for low-risk women and focusing colposcopy capacity on those most likely to benefit (Gisca et al., 2025; Bautista et al., 2025). Within the NHS cervical screening programme, colposcopy services represent a constrained resource, and current HPV-based triage generates substantial volumes of referrals for women whose infections would otherwise regress, underscoring the need for more precise risk stratification to make more efficient use of colposcopy capacity.

## 3 Vaginal microbiome and determinants

The vaginal microbiome is a complex ecosystem central to women’s reproductive health, with direct implications for infection susceptibility, immune modulation, HPV persistence, and cervical carcinogenesis (Bautista et al., 2025; Alimena et al., 2022). It comprises bacteria, fungi, and viruses residing in the vaginal tract; in healthy states, *Lactobacillus* species predominate and produce lactic acid, maintaining an acidic pH (≤4.5) that supports mucosal integrity and provides defence against invading pathogens (Kalia et al., 2020; Avitabile et al., 2024). Disruption of this eubiotic state leads to more diverse, anaerobe-rich communities (dysbiosis or non-*Lactobacillus*-dominant states) that are consistently associated with increased susceptibility to genital infections, higher rates of persistent high-risk HPV, and elevated risk of cervical intraepithelial neoplasia and cancer (Kazlauskaitė et al., 2025; Rinninella et al., 2025).

The vaginal microbiome is frequently classified into community state types (CSTs), which capture dominant taxa and their clinical correlates (Table 1). CST I, II and V are characterised by dominance of *L. crispatus*, *Lactobacillus gasseri* and *Lactobacillus jensenii*, respectively, and are generally considered protective, whereas CST III, dominated by *L. iners*, represents a more unstable, transitional state that is prone to shift towards dysbiosis (Zheng et al., 2021). CST IV encompasses heterogeneous, high-diversity communities enriched for anaerobic pathobionts and is associated with higher vaginal pH, bacterial vaginosis and increased risk of HPV persistence and cervical neoplasia (Bautista et al., 2025). The CST I–IV nomenclature derives from foundational work by Ravel and colleagues and has since been validated in longitudinal studies and meta-analyses across diverse populations (Ma and Li, 2017). *Candida* species are not included in standard CST classification, but vulvovaginal candidiasis can coexist with both *Lactobacillus*-dominated and dysbiotic communities and often coincides with shifts away from stable *Lactobacillus* dominance (Tortelli et al., 2020). Quantifiable ecological markers such as CST assignment and vaginal pH therefore represent attractive, low-dimensional candidates for incorporation into predictive algorithms.

**TABLE 1:** Community state types (CSTs) (De Seta et al., 2019).

CST type	Dominant bacterium	pH	Clinical association
I	*L. crispatus*	≤4.5	Most protective; promotes vaginal health
II	*L. gasseri*	≤4.5	Generally healthy, less robust than CST I
III	*L. iners*	Variable	Transitional; more prone to dysbiosis
IV	Anaerobes (*Gardnerella*, etc.)	>4.5	Dysbiotic; higher risk of BV, HPV, and cervical neoplasia
V	*L. jensenii*	≤4.5	Rare, but considered protective

The composition and stability of the vaginal microbiome are shaped by multiple intrinsic and extrinsic determinants, including lifestyle exposures, medical interventions, hormonal status and life stage (A et al., 2021). Smoking reduces *Lactobacillus* abundance and increases bacterial vaginosis risk through anti-estrogenic effects and exposure to toxic metabolites such as benzo [a]pyrene diol epoxide, which together disrupt protective communities and impair local immune function (Pavlova and Tao, 2000). Genital tract inflammation, whether driven by sexually transmitted infections, non-specific cervicitis, immune dysregulation or irritants, perturbs mucosal barriers and favours overgrowth of anaerobic pathobionts (Smritee et al., 2021). Antibiotic use, particularly repeated or broad-spectrum courses, can deplete *Lactobacillus* species, lower colonisation resistance and precipitate recurrent bacterial vaginosis or candidiasis (Muzny and Sobel, 2022). Sexual behaviour, including number of partners and inconsistent condom use, is associated with greater temporal variability and diversity of the microbiome, partly via introduction of exogenous organisms and inflammation (Wessels et al., 2017). Exogenous hormones also modulate community structure: some hormonal contraceptives appear to stabilise *Lactobacillus*-dominant states, whereas others may increase microbial diversity, depending on formulation and individual response (Tuddenham et al., 2023). Age and menopausal status exert strong effects; postmenopausal women typically display reduced *Lactobacillus* dominance and higher pH, while adolescence and lower reproductive age are associated with higher *Lactobacillus* prevalence but also dynamic fluctuations around menarche, menstruation, pregnancy and postpartum transitions (Park et al., 2023; Chen et al., 2025). Many of these determinants are routinely captured in clinical practice and can be incorporated as features or interaction terms in predictive models, enabling algorithms to learn how microbiome profiles and host or behavioural factors jointly influence HPV persistence and progression risk.

Beyond oncology, vaginal microbiome assessment is already being explored in several clinical domains, underscoring its broader translational potential. In fertility and *In-vitro* Fertilisation (IVF) settings, aberrant communities with depletion of *Lactobacillus* and overrepresentation of anaerobic pathobionts have been linked to reduced implantation rates and early pregnancy loss, and early interventional studies suggest that targeted antibiotic–probiotic strategies and restoration of *Lactobacillus crispatus* dominance may improve outcomes (Väinämö et al., 2023). In the management of recurrent bacterial vaginosis, vulvovaginal candidiasis and sexually transmitted infections, microbiome profiling is increasingly used to guide therapy and reduce recurrence (Grando and Watson, 2025). Emerging data also support roles in predicting or managing preterm birth, menopausal symptoms and certain gynaecological conditions such as endometriosis and pelvic inflammatory disease, although routine use remains limited (Sofia et al., 2025). Despite this broadening evidence base, large-scale clinical adoption of vaginal microbiome testing in public health systems is rare, and the NHS does not currently screen or intervene based on microbiome profiles (Sofia et al., 2025; Medic ines and Healthcare products Regulatory Agency MHRA, 2025). This reflects workflow, regulatory and evidentiary gaps but also represents a clear opportunity to embed validated microbiome-informed tools into screening and preventive pathways.

From a cervical cancer perspective, integration of microbiome signatures into prediction models remains particularly sparse. Systematic reviews and multi-omics studies consistently show that non-*Lactobacillus*-dominated or *L. iners*-dominated communities, together with anaerobic pathobiont enrichment, are associated with higher odds of high-risk HPV persistence and progression to CIN2/3, whereas *L. crispatus*-dominated communities are protective (Dai et al., 2021). However, most existing studies are cross-sectional, with limited adjustment for behavioural and demographic confounders, and there is a paucity of large, ethnically diverse, longitudinal cohorts, especially within the United Kingdom, suitable for training and validating predictive algorithms. Recent work at the interface of host epigenetics and the microbiome suggests that combined cervicovaginal methylation and microbial signatures may enhance risk discrimination. For example, a study reported that panels of host DNA methylation markers measured on cervical samples, when analysed together with CST status and enrichment of anaerobic taxa, improved separation of CIN2+ from ≤CIN1 compared with methylation or microbiome features alone, with area-under-the-curve values typically in the 0.80–0.90 range in small case–control cohorts (Nené et al., 2020). Although these dual biomarkers remain in early development and have not yet been tested in large, prospective screening populations, they illustrate how epigenetic and microbiome layers can be co-modelled and motivate their inclusion as candidate features in next-generation AI risk engines (Sahoo et al., 2025). Collectively, the determinants and modifiers of the vaginal microbiome generate substantial inter-individual variation in infection risk and vulnerability to neoplastic progression, yet these data are almost entirely absent from current cervical cancer risk calculators and NHS screening pathways. This provides a natural, immediately exploitable feature space for AI-driven risk models, in which CST assignment, relative abundance of key taxa (for example, *L. crispatus*, *L. iners*, *Gardnerella*), and simple ecological indices (such as pH or diversity metrics) can be combined with HPV genotype and clinical covariates in supervised learning frameworks.

## 4 Mechanistic interactions between HPV and the microbiota

The mechanistic interplay between the vaginal microbiota and HPV persistence is increasingly recognised as central to cervical carcinogenesis. As outlined in previous sections, dominant *Lactobacillu*s communities, particularly those enriched in *L. crispatus*, maintain a low vaginal pH, preserve epithelial barrier integrity, and modulate local immune responses in ways that favour viral clearance and tissue homeostasis (Bautista et al., 2025). In contrast, a shift towards anaerobic pathobiont-rich communities (for example, *Gardnerella, Atopobium* and related taxa) raises vaginal pH, increases mucosal inflammation, and disrupts epithelial structure, thereby weakening immune surveillance and creating a permissive niche for persistent infection with oncogenic HPV types (France et al., 2022). Mechanistic and multi-omic studies implicate a convergent set of pathways in this transition, including increased production of pro-inflammatory cytokines, upregulation of immune evasion markers, and metabolic disruption such as depletion of lactic acid and increased proteolytic and genotoxic activity, which together impair effective antiviral responses and promote cellular instability (Marrocco and Ortiz, 2022). Loss of *L. crispatus*–mediated protection, coupled with expansion of anaerobic taxa, therefore drives a causal chain of higher pH, chronic inflammation and impaired clearance, which accelerates progression from HPV infection to cervical intraepithelial neoplasia and, in a minority of women, invasive cancer (Avsaroglu et al., 2023). Pilot interventional studies targeting restoration of *Lactobacillus* dominance, notably with intravaginal *L. crispatus*–based probiotics, provide preliminary support for microbiome modulation as a strategy to reduce HPV-related risk (Liu et al., 2024). In small, single-arm or randomised trials, women with persistent high-risk HPV or low-grade CIN who received *L. crispatus* preparations showed higher rates of HPV clearance (often in the range of 40%–60% at 6–12 months) and reduced recurrence of high-grade CIN compared with historical or concurrent controls, alongside durable shifts from CST IV/III towards CST I–like communities in a subset of participants (Schellekens et al., 2025; Wu et al., 2024). These studies are heterogeneous, underpowered and limited by short follow-up, but they suggest that correcting dysbiosis and re-establishing *L. crispatus*–dominant states is biologically plausible and may be clinically meaningful, warranting larger, rigorously designed randomised trials with virological and histological endpoints.

## 5 Methodological advances and evidence gaps

Recent microbiome studies in HPV and cervical cancer have benefited from substantial methodological advances. Early work relied on 16 S rRNA gene sequencing to describe vaginal community state types, linking loss of *Lactobacillus* dominance and increased microbial diversity to greater HPV persistence and dysplasia risk, but this approach provided only broad bacterial profiles and largely missed non-bacterial taxa and functional pathways (Shen-Gunther et al., 2025; Osei Sekyere et al., 2025). Shotgun metagenomics and metatranscriptomics now enable more detailed characterisation of viruses, fungi, metabolic functions, and host–microbe interactions, revealing how microbial richness and functional capacity vary by HPV genotype and cancer stage, albeit at the cost of increased technical variability related to sampling, sequencing platforms, and bioinformatic pipelines (Bautista et al., 2025; Aitmanaitė et al., 2023). Most published studies remain observational and cross-sectional, so although they report strong associations, causal inference is limited by confounding from sexual behaviour, contraceptive use, host genetics, and ethnicity, together with under-representation of United Kingdom and other multi-ethnic screening populations. These limitations correspond to substantial concerns across multiple PROBAST domains, including participant selection, predictor measurement, outcome definition, and analysis. Many published AI and risk-score studies mix case–control and cohort designs, use convenience samples, or apply *post hoc* predictor selection without appropriate shrinkage, all of which can inflate apparent performance and reduce generalisability. Future microbiome-enabled models should therefore be prospectively specified, developed in representative screening cohorts, and reported with transparent PROBAST-aligned risk-of-bias assessments.

The scarcity of longitudinal datasets restricts the ability to track dynamic changes in the cervicovaginal microbiome before, during, and after persistent high-risk HPV infection or progression to high-grade lesions (Jung et al., 2025). Addressing these gaps will require prospective, longitudinal multi-omic cohorts, ideally multi-ethnic and UK-based, in which HPV primary screening is combined with predefined microbiome measurements on the same sample, for example, CST classification; relative abundance or load of key taxa such as *L. iners, L. crispatus, Gardnerella* and *Atopobium*; and simple ecological metrics (pH, diversity indices), plus linked virome, epigenetic and immune readouts (for example, targeted methylation panels and immunophenotyping) (Zeng et al., 2023). Such datasets are needed to define dynamic risk signatures, quantify how specific microbial patterns modify progression probabilities given HPV genotype and host factors, and support robust biomarker discovery and validation for future cervical cancer risk models.

## 6 Implications for predictive modelling and clinical translation

Thus far, some AI models have been developed to assist in the prediction of clinical outcomes in relation to cervical cancer. These models typically draw on demographic, clinical, and imaging variables (features), and encompass an array of traditional statistical approaches and modern DNNs. For instance, Matsuo et al. (2019), Yu et al. (2022), Kolasseri et al. (2024) and Pu et al. (2025) employed demographic and clinical variables to predict the survival outcomes (Mats et al., 2019; Yu et al., 2022; Kolasseri and B, 2024; Pu et al., 2025). It is worth noting that most approaches used to date rely on traditional statistical models. This is largely because the input variables do not involve complex spatial patterns (e.g., images) in the prognostic context, meaning that classic statistical methods may perform just as well, if not better, than DNNs (Eloranta and Boman, 2022). Some statistical models such as logistic regression, also possess the strength of interpretability, as the association (and statistical significance) between the variables and the outcomes can be obtained. Bao et al. (2020), Hunt et al. (2021), Park et al. (2021) and Yang et al. (2025) leveraged specialised imaging modalities to distinguish cervical cancer from other types (Bao et al., 2020; Hunt et al., 2021; Park et al., 2021; Yang et al., 2025). Since this line of research takes the medical images as the input to the AI models, CNNs, a specific type of DNNs, may play a more crucial role in prediction. CNNs can automatically learn useful features from raw data such as complex patterns. This makes them especially powerful for imaging tasks, whereas statistical models rely on handcrafted variables. Bao et al. (2020), Hunt et al. (2021), Namalinzi et al. (2024) and Khiruddin et al. (2025) also tried to predict the lesion types which may be of direct relevance in the screening context (Bao et al., 2020; Hunt et al., 2021; Namalinzi et al., 2024; Khiruddin et al., 2025). A more detailed breakdown of these papers are shown in the Table 2 below. Here we do not aim to provide an exhaustive list of published papers but instead, to shed light on the latest development and the AI models used for these tasks.

**TABLE 2:** Papers demonstrating latest developments in AI models for predictive algorithms for cervical cancer.

Citation	Year	ML method(s)	Features/predictor variables	Targets/outcome(s)	Clinical context
Matsuo K et al., Am J obstet gynecol	2019	DNNs (fully connected feedforward) vs. Statistical models (CoxBoost, CoxLasso, random survival forest)	40 features set 1: demographics set 2: histologics (tumour characteristics) set 3: treatment	Survival time prediction of cancer patients	Prognosis
Bao H et al., gynecol oncol	2020	CNNs	digital cytological images	Negative CIN1, CIN2, CIN3, cervical cancer. Evaluation of binary cutoff such as < CIN2 vs. CIN2+	Screening/Diagnosis
Hunt B et al., int J cancer	2021	Multi-task CNNs (segmentation and classification)	High-resolution microendoscopy (HRME)	Negative CIN1, CIN2, CIN3, invasive carcinoma. Evaluation of binary cutoff such as < CIN2 vs. CIN2+	Screening/Diagnosis
Park YR et al., sci rep	2021	CNNs (using the whole image) vs. statistical models (XGBoost, SVM, RF)	Cervicography images for CNNs 10 extracted features selected from over 300 b y LASSO for statistical models	Negative vs. cervical cancer	Diagnosis
Yu W et al., cancer med	2023	LR, RF, SVM, XGBoost	7 clinical factors selected by LASSO	5-year survival status of nonmetastatic cervical cancer patients	Prognosis
Namalinzi F et al., BMC womens health	2024	Lr, Rf, SVM, KNN, MLP	5 out of 16 socio-demographic characteristics and clinical factors were selected using LR and RFERF	Precancerous cervical cancer lesions	Screening
Kolasseri AE et al., sci rep	2024	Cox model, weibull model, random survival forest	10 demographic and clinical variables	Survival time prediction of cancer patients	Prognosis
Khiruddin K et al., PLoS one	2025	CNNs	Pap smear images	3 categories: Normal, LSIL, HSIL	Screening
Pu Y et al., sci rep	2025	GBSA, RSF, ST, CoxPH, and CoxTV	6 selected from 27 demographics,tumor characteristics, and treatment modalities	5-year survival prediction	Prognosis
Yang WJ et al., sci rep	2025	LR, SVM, RF, XGBoost	Cervicographic images with acetowhite areas	Atypical vs. cervical cancer	Screening/Diagnosis

In a screening setting, a “clinically meaningful” risk calculator would operate at the point of screening or early follow-up to stratify asymptomatic or mildly symptomatic women with HPV positivity or equivocal cytology into clearly actionable groups, such as “return to routine recall”, “repeat testing at shorter interval”, or “direct referral to colposcopy” (Wentzensen et al., 2016). It would need to integrate variables already available or readily collectable in screening workflows (for example, HPV genotype, age, smoking status, parity, contraceptive use and prior screening history), together with vaginal microbiome summaries such as community state type, relative abundance of *L. crispatus* and anaerobic pathobionts, and simple ecological measures (for example, diversity or pH) (Qi et al., 2024). Where available, immune or inflammatory markers could be included as additional predictors, such as cervicovaginal cytokine panels (e.g., interleukin-1β, interleukin-6, TNFα), measures of mucosal antibody responses, or systemic indicators of immunosuppression (Park et al., 2020). From a computational perspective, these microbiome, immune and lifestyle features can be added to existing tabular models (for example, penalised logistic regression, random forests, gradient boosting) without major architectural changes, while maintaining interpretability through odds ratios, hazard ratios or feature importance measures; for image-rich settings, CNNs or vision transformers can simply be extended with additional neurons that ingest microbiome and clinical covariates alongside image embeddings (Γεώργιος et al., 2023).

Proof-of-concept studies could therefore begin as soon as robust, standardised microbiome measurements are available, for example, through 16 S rRNA or shotgun sequencing processed via harmonised pipelines and reported as a small number of clinically interpretable features (such as CST, *L. crispatus* abundance and a binary dysbiosis index) (Asensio-Puig et al., 2024). In a pragmatic NHS workflow, self-collected tampon samples or clinician-taken swabs would be processed in existing accredited laboratories, with microbiome and HPV results returning within the same time frame as current HPV tests (typically a few days), and fed into an AI risk engine integrated with laboratory and GP systems. Initial deployment should prioritise cost-conscious designs that use relatively low-cost sequencing or targeted panels, and focus on clear risk thresholds rather than complex continuous outputs, to support straightforward clinical interpretation. Early phase studies will need to collect detailed implementation data on turnaround times, incremental laboratory costs, interpretability for clinicians, and patient acceptability, and should be designed to test whether microbiome-informed risk scores actually reduce unnecessary colposcopy referrals and follow-up visits while maintaining or improving detection of high-grade lesions. In this way, vaginal microbiome-informed modelling becomes a practical extension of current AI approaches rather than a complete paradigm shift, while addressing a key translational gap and aligning with regulatory expectations for external validation, bias assessment and transparent model behaviour in NHS-relevant screening and triage populations.

## 7 Validation, harmonisation and explainability

Validation of microbiome-informed prediction models should be explicitly staged, moving from rigorous internal cross-validation to external testing in United Kingdom and European cohorts that mirror real NHS screening populations. Performance must be stress-tested across centres, laboratory platforms, and demographic strata, with pre-specified sub-analyses for HPV-positive/cytology-negative women and under-screened communities and ultimately evaluated prospectively within existing screening workflows to capture operational failure modes, data missingness, and clinician behaviour (Yin et al., 2025). Given the variability in sequencing pipelines, sample handling, and IT infrastructures, systematic harmonisation is essential: batch-effect correction tools such as ComBat and related methods, widely used in radiomics and transcriptomics, should be adapted and validated for microbiome and HPV sequencing data to support robust cross-laboratory deployment (Yu et al., 2024). In parallel, explainability must be built into the model lifecycle, with routine use of feature-attribution techniques (for example, SHAP for tabular and omic inputs, Grad-CAM or analogous methods for imaging) and global model summaries, together with predefined thresholds for acceptable performance degradation in external cohorts and transparent reporting of subgroup results (Hettikankanamage et al., 2025). Bias audits covering ethnicity, age, deprivation, proxies for sexual behaviour, and HPV vaccination status should be embedded as a core requirement rather than an optional add-on, ensuring that microbiome-enabled tools are both technically sound and equitable in NHS and international settings.

## 8 Integration into NHS screening pathways

Integration of vaginal microbiome–informed algorithms into the NHS cervical screening pathway offers a realistic route to more personalised risk stratification and more efficient use of colposcopy capacity. In a feasible workflow, both clinician-collected and self-collected cervico-vaginal samples could be processed using standardised protocols to generate concurrent HPV genotype and microbiome profiles in accredited laboratories, with results automatically ingested by laboratory and GP information systems. An AI risk engine would then compute a composite risk score that integrates HPV status, microbiome community state, vaccination history and key clinical factors, routing women with persistent high-risk HPV and dysbiotic microbiome signatures directly to colposcopy while reassuring low-risk individuals and returning them to routine recall, thereby reducing unnecessary referrals, anxiety and downstream overtreatment. Detecting a defective or dysbiotic microbiome within this pathway also creates actionable levers for risk reduction, because women identified as high risk could be offered targeted interventions to restore *Lactobacillus*-dominant communities, such as *L. crispatus*–based probiotics, behavioural modification (for example, smoking cessation or contraceptive review), or tailored antibiotic–probiotic regimens alongside closer surveillance, with emerging trial data suggesting that such strategies can enhance HPV clearance and reduce recurrence of high-grade lesions. However, several barriers must be anticipated: microbiome testing is not currently embedded in NHS cervical screening protocols, most laboratories lack validated pipelines or LIMS fields for these data, and workflows are optimised around binary HPV and cytology results with established quality metrics (Gillibrand et al., 2025). Early pilots should therefore embed microbiome sampling and algorithmic scoring within existing structures, leveraging assets such as the NHS App, electronic cytology platforms, and AI-enabled referral tools already evaluated in other cancer pathways, so that these pilots can act as testbeds for interoperability standards, consent models, data security requirements, operational key performance indicators, and formal health economic evaluation comparing HPV-only versus multimodal strategies. Because HPV primary screening already relies on high-throughput molecular testing of cervicovaginal samples, integrating vaginal microbiome profiling would not require additional sample collection, only an incremental analytical step on the same self-collected or clinician-collected specimen (Gupta et al., 2018). In a phased implementation, microbiome analysis could initially be performed in batch on HPV-positive samples using existing liquid-based cytology or swab/tampon eluates, with bioinformatic processing and risk scoring aligned to current HPV laboratory turnaround times (typically 1–2 weeks from sample receipt to GP notification), so that microbiome-informed risk estimates can be returned alongside HPV results without delaying recall or referral decisions. This design keeps marginal costs largely confined to sequencing or targeted panel assays and informatics, while preserving the familiar cadence of screening for women and providers.

Although we focus on the NHS pathway, the same microbiome-enabled risk-modelling framework could be adapted for use in LMIC screening programmes that increasingly rely on HPV self-sampling, community-based outreach, and screen-and-treat approaches. In these settings, cost-conscious designs, such as targeted testing among HPV-positive self-samplers, simplified microbiome signatures, and integration with existing digital registries, will be essential to ensure that AI-assisted triage improves screening uptake and is at least cost-neutral relative to clinician-collected strategies, as suggested by emerging LMIC self-sampling cost studies.

## 9 Future directions and unmet needs

**FIGURE 1:**
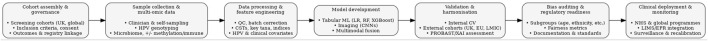
Proposed end‐to‐end pipeline for microbiome‐informed AI risk modelling in cervical screening, spanning cohort assembly, multi‐omic data generation, feature engineering, model development, staged validation, bias auditing and deployment within routine screening pathways.

Future work should prioritise establishment of large, prospective, multi-omic and multi-ethnic cohorts that combine self-sampling, detailed behavioural and vaccination data, and longitudinal outcomes across multiple screening rounds, with sufficient power to examine intersectional disparities and to train temporal models (for example, Bayesian change-point methods, recurrent networks, or transformer-based architectures) that capture dynamic changes in microbiome composition, HPV status, and host factors over time. These cohorts should be complemented by randomised or pragmatic real-world evaluations of algorithm-guided pathways, such as modified recall intervals, targeted microbiome-restoring interventions, and tailored digital risk communication, to quantify effects on uptake, adherence, equity, and over- or under-treatment. At the same time, governance frameworks for bias auditing, transparency, and reporting need to be aligned with NHS, WHO, and emerging global AI standards, including standardised model cards, explicit documentation of training and validation datasets, and shared benchmarks for fairness and performance across demographic and geographic subgroups. Embedding microbiome discovery and predictive modelling within a network-physiology framework that links viral dynamics, host immunity, vaginal ecology, vaccination status, behaviour and social determinants, and validating the resulting tools in United Kingdom/NHS and international settings, will be essential to deliver microbiome-enabled cervical screening that is personalised, equitable and regulation-ready. Figure 1 outlines a proposed end-to-end pipeline from cohort assembly and multi-omic data generation through feature engineering, model development, staged validation and bias auditing to clinical deployment within NHS and other screening pathways.

## Data Availability

The original contributions presented in the study are included in the article/supplementary material, further inquiries can be directed to the corresponding author.
